# Supplementary material for: Acoustic Atomization-Induced Pumping Based on a Vibrating Sharp-Tip Capillary
Source: Micromachines (Basel). 2023 Jun 8;14(6):1212. doi: 10.3390/mi14061212 (PMC10301781; doi:10.3390/mi14061212)
Supplement: Supplementary file 1 [file micromachines-14-01212-s001.zip › micromachines-2416728 Supporting information.pdf]

Supporting Information

Acoustic atomization induced pumping based on a vibrating sharp-tip capillary

Balapuwaduge Lihini Mendis[1], Ziyi He[2], Xiaojun Li[1], Jing Wang[1], Chong Li[1], Peng Li[1]*

1. C. Eugene Bennett Department of Chemistry, West Virginia University, Morgantown, WV, USA.
2. College of Veterinary Medicine, Huazhong Agricultural University, Wuhan, Hubei 430070, China.

* Correspondence and requests for materials should be addressed to P.L. (peng.li@mail.wvu.edu)

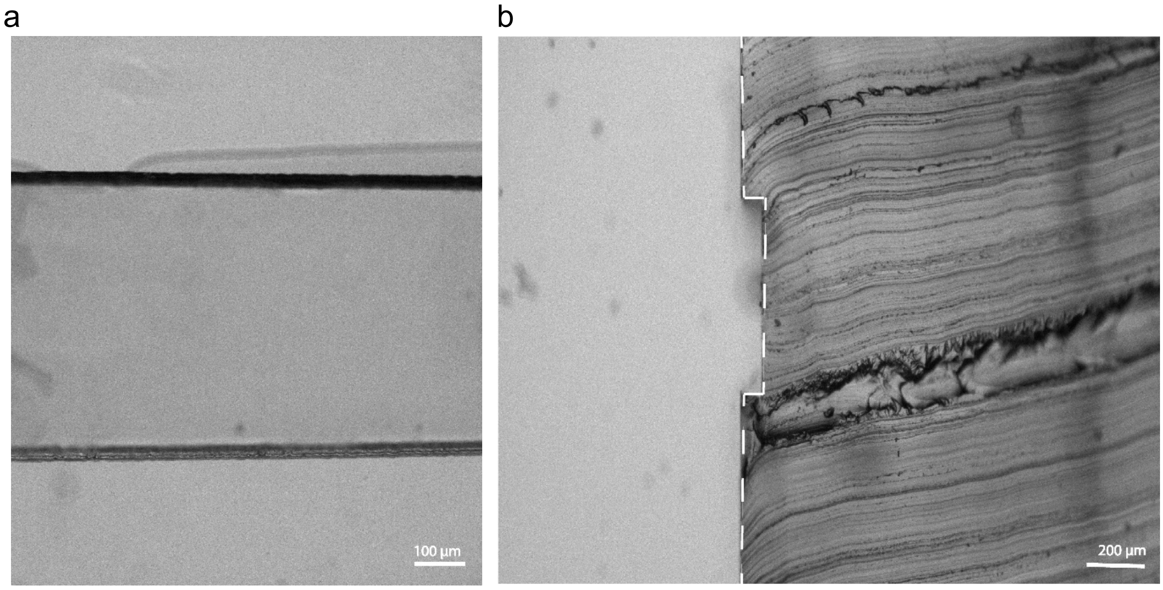

Figure S1. Microscopic images of the PDMS microchannel. a) overview of the channel showing a width of 600 µm. b) cross section the channel showing a height of 90 µm.

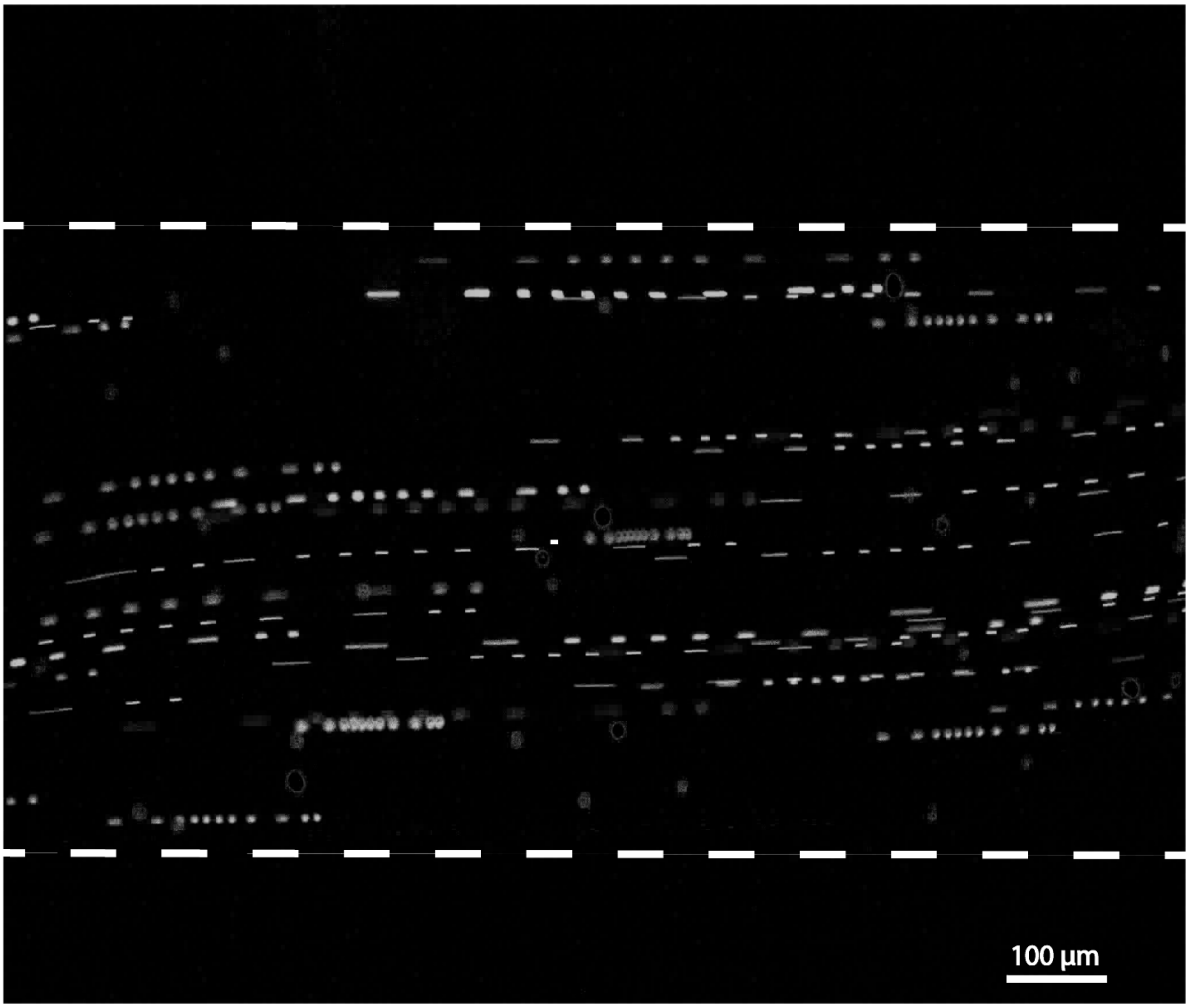

Figure S2. Stacked images of showing beads movement in a PDMS microchannel with a flow rate of ~5 µL/min.
